# e-Health Strategy for Surgical Prioritization: A Methodology Based on Digital Twins and Reinforcement Learning

**DOI:** 10.3390/bioengineering12060605

**Published:** 2025-06-02

**Authors:** Fabián Silva-Aravena, Jenny Morales, Manoj Jayabalan

**Affiliations:** 1 Facultad de Ciencias Sociales y Económicas, Universidad Católica del Maule, Avenida San Miguel 3605, Talca 3460000, Chile; jmoralesb@ucm.cl; 2 School of Design, Bath Spa University, Bath BA2 9BN, UK; m.jayabalan@bathspa.ac.uk

**Keywords:** e-Health platform, intelligent scheduling, Digital Twin, reinforcement learning, equitable access, digital decision support

## Abstract

This article presents a methodological framework for elective surgery scheduling based on the integration of patient-specific Digital Twins (DTs) and reinforcement learning (RL). The proposed approach aims to support the future development of an intelligent e-health platform for dynamic, data-driven prioritization of surgical patients. We generate prioritization scores by modeling clinical, economic, behavioral, and social variables in real time and optimize access through a reinforcement learning engine designed to maximize long-term system performance. The methodology is designed as a modular, transparent, and interoperable digital decision-support architecture aligned with the goals of organizational transformation and equitable healthcare delivery. To validate its potential, we simulate realistic surgical scheduling scenarios using synthetic patient data. Results demonstrate substantial improvements compared withto traditional strategies, including a 55.1% reduction in average wait time, a 41.9% decrease in clinical risk at surgery, a 16.1% increase in OR utilization, and a significant increase in the prioritization of socially vulnerable patients. These findings highlight the value of the proposed framework as a foundation for future smart healthcare platforms that support transparent, adaptive, and ethically aligned decision-making in surgical scheduling.

## 1. Introduction

Managing surgical waitlists in public health systems represents a pressing challenge at the intersection of clinical, operational, and ethical priorities [1,2]. Elective surgeries often face extended delays due to limited OR capacity, unpredictable patient flow, and administrative bottlenecks [3,4,5]. These delays can lead to deteriorating health outcomes, increased morbidity, and dissatisfaction among patients and providers alike [6]. The situation is further complicated by the increasing volume of cases, aging populations, and the need to balance efficiency with fairness [7,8].

Traditional scheduling methods, such as First-Come-First-Served (FCFS) or categorical risk-based prioritization, fail to account for the dynamic nature of patient conditions [9]. These methods often overlook the socioeconomic context of individuals, changes in clinical urgency over time, and the system-wide impact of inefficient planning [10]. In addition, static models cannot respond to fluctuating institutional capacities or simulate the future state of patient trajectories, which limits their ability to optimize long-term outcomes across a heterogeneous population [11].

Recent advances in artificial intelligence (AI), digital health platforms, and simulation modeling open new pathways to transform the way we prioritize surgical care [12,13]. Digital Twin technologies allow real-time modeling of patient evolution, capturing changes in clinical risk, behavioral engagement, and vulnerability [14,15,16]. Reinforcement learning, in parallel, enables systems to learn optimal decision policies by interacting with an environment and maximizing future rewards [17,18,19]. Although these two technologies have individually gained traction in the healthcare and engineering domains, their combined application to surgical scheduling remains underexplored.

In light of these limitations, there is a clear methodological gap in current surgical scheduling systems: Most frameworks remain static, unidimensional, or unclear in their decision logic. Few approaches provide a transparent, ethically configurable, and dynamically adaptive mechanism to prioritize patients based on real-time clinical deterioration, equity concerns, and resource constraints. Moreover, the integration of predictive modeling with learning-based optimization remains underexplored, particularly in public hospital settings where transparency and fairness are critical.

In this work, we propose a simulation-based methodology that addresses the limitations of traditional surgical scheduling by integrating patient-specific DT modeling with RL. Our framework generates multidimensional prioritization scores that evolve over time, capturing clinical risk, social vulnerability, satisfaction signals, and economic value, and uses these scores to train an RL agent capable of optimizing scheduling decisions under real-world constraints. The methodology is embedded in a conceptual e-health ecosystem designed for transparency, adaptability, and ethical alignment, paving the way for future deployment in intelligent, digitally integrated surgical planning platforms.

DTs enable us to simulate the temporal evolution of individual patient states, allowing personalized anticipation of clinical deterioration, behavioral disengagement, and social vulnerability, dimensions often overlooked in static scheduling models. RL, in turn, enables adaptive policy optimization in environments where decision trade-offs (e.g., efficiency vs. equity) must be learned and balanced dynamically. Together, these technologies directly address the core limitations identified in current scheduling systems: lack of responsiveness, transparency, and ethical adaptability.

The paper is organized as follows. First, in Section 2, we review the relevant literature on surgical scheduling, reinforcement learning, and Digital Twin applications in healthcare. In Section 3, we then describe our methodological framework in detail, followed by a simulation-based evaluation that compares the proposed system with traditional scheduling strategies. Section 4, we present the results. Then, in Section 5, we discuss implications and limitations, and in Section 6, we conclude with future research directions.

## 2. Literature Review

The literature on surgical scheduling has traditionally focused on operations research and mathematical optimization models [20,21]. Integer linear programming, mixed integer scheduling, and simulation-based heuristics have been widely used to optimize operating room allocation and patient throughput [22]. These models often assume static patient characteristics and deterministic environments, limiting their capacity to address uncertainty and patient heterogeneity. The foundational works of [23,24] offer valuable frameworks but primarily prioritize resource utilization, not evolving patient risk.

RL has gained attention for its capacity to optimize sequential decisions under uncertainty [25]. In healthcare, RL has been applied to problems such as patient flow management (see, e.g., [19,26]), resource allocation, such as [19], and personalized care pathways, such as [27]. Although promising, most RL applications focus on operational efficiency rather than equity or patient-centered prioritization [28]. In addition, few implementations incorporate dynamic clinical variables or patient-specific simulations in real time.

DT technology has emerged as a key innovation in personalized medicine [14,29]. It has been applied to monitor and predict patient outcomes in cardiology, oncology, and intensive care, often using real-time data from wearables and monitoring systems [30,31]. Studies by [32,33] demonstrate their value in capturing temporal changes in patient status. However, the integration of Digital Twins into operational hospital decision-making, particularly for surgery scheduling, remains limited.

Recent contributions, such as [34] and others, have begun to explore hybrid models that combine AI with operational research techniques. For example, some studies, such as [35,36] have investigated the use of machine learning to classify surgical urgency or predict cancellations. However, the integration of reinforcement learning with multidimensional patient modeling, such as Digital Twins, has not been thoroughly investigated for equitable surgical prioritization.

Our work advances the literature by proposing a holistic, simulation-validated methodology that fuses Digital Twin modeling with RL-driven decision-making. Unlike previous studies, our framework explicitly incorporates ethical dimensions such as fairness in access for vulnerable populations while also ensuring operational efficiency and clinical safety. In addition, it contributes to the evolution of digital platform design in the healthcare domain, offering a user-centered, intelligent service architecture that supports transparent and adaptive prioritization. This positions our contribution within the broader scope of applied electronic commerce in health services, expanding the conceptual boundaries of AI-enabled e-health innovation.

## 3. Methodology

In this section, we present a dynamic and explainable methodology for prioritizing elective surgical patients through the integration of technology, DT, RL, and e-health decision ecosystems. Our approach is structured into five steps, each contributing to the construction of an intelligent, ethical, and digitally embedded scheduling system.

We designed this methodological architecture specifically to overcome the limitations of current surgical scheduling systems. DTs allow us to model multidimensional patient states in real time, including clinical, behavioral, social, and economic aspects, while RL enables the system to learn dynamic prioritization strategies that optimize clinical safety, operational efficiency, and equity. This synergy directly addresses the need for adaptability, explainability, and fairness in prioritization frameworks for public hospital environments.

### 3.1. Step 1: System Overview and Dataset Specification

We begin by defining $P=\{p_{1},p_{2},\ldots ,p_{n}\}$ as the finite set of patients currently registered on the elective surgical waiting list. For simulation purposes, we generated a synthetic cohort of n=1000 patients based on clinically informed distributions aligned with public hospital settings. This sample size was chosen to ensure adequate variability in patient attributes while maintaining computational tractability. Each individual patient $p_{i}\in P$ is characterized at the time of registration by a multidimensional attribute vector $x_{i}$, which includes demographic, clinical, economic, and psychosocial components. Specifically,

(1) $$\begin{array}{c} x_{i}=\left(a_{i},g_{i},d_{i},r_{i0},w_{i0},e_{i},s_{i},v_{i}\right) \end{array}$$

where:

- $a_{i}$: Age of patient $p_{i}$ at time of registration (years);
- $g_{i}$: Gender, encoded as a binary or categorical variable;
- $d_{i}$: Primary diagnosis or procedure code;
- $r_{i0}$: Initial clinical risk score assigned by medical staff;
- $w_{i0}$: Time already spent on the waiting list (in days or weeks);
- $e_{i}$: Expected economic value or reimbursement associated with the intervention (e.g., DRG-based revenue);
- $s_{i}$: Initial satisfaction or engagement score based on digital platform interactions;
- $v_{i}$: Vulnerability index that captures socioeconomic and psychosocial risk (as proposed by [37,38,39,40]).

These patient characteristics come from a unified data ecosystem that combines hospital electronic health records (EHRs), patient-reported outcomes, behavioral app data, and socioeconomic profiles. The richness and diversity of the data allow us to build a robust digital representation of each patient, ideal for subsequent simulation and prioritization.

To ensure clinical plausibility, we modeled the system based on elective surgeries within a high-demand specialty of otolaryngology (ENT). This domain was selected due to its typical waiting list characteristics, diversity of procedures, and sensitivity to scheduling delays. The simulation parameters reflect patterns commonly observed in ENT services from public hospitals.

To manage a design of this nature, we propose that this system be scalable and modular. To this end, we organize the architecture into three distinct but interconnected layers:

- Data layer: Responsible for collecting, cleaning, integrating and storing heterogeneous sources of patient information, including clinical diagnostics, monitoring outputs, digital engagement records, and financial metadata.
- Digital twin layer: Creates and continuously updates a real-time digital representation of each patient, reflecting their evolving health status, economic profile, satisfaction signals, and social vulnerability. These Digital Twins form the core analytic object used in decision-making.
- Decision layer: Implements intelligent scheduling decisions through a combination of prioritization logic and reinforcement learning algorithms. Select patients dynamically according to multiple and potentially conflicting criteria.

This modular framework ensures flexibility in deployment and interpretability in operation. Each component feeds the next in a feedback-informed pipeline. The current step sets the foundation for all subsequent layers of modeling by formalizing the patient data model and defining the multidimensional nature of prioritization.

### 3.2. Step 2: Digital Twin Modeling of Surgical Patients

Building on the multidimensional representation of patients defined in Step 1, we now construct a DT model for each patient $p_{i}\in P$. A DT is a virtual proxy that evolves over time and captures the real-time status and the projected evolution of the patient’s condition [29,30,31]. Synthesizes clinical risk, economic impact, digital behavior, delay-related penalties, and vulnerability into a unified temporal model.

Formally and similar to [15], we define the DT of the patient $p_{i}$ at time *t* as

(2) $$\begin{array}{c} DT_{i}\left(t\right)=\left[R_{i}\left(t\right),E_{i}\left(t\right),S_{i}\left(t\right),D_{i}\left(t\right),V_{i}\left(t\right)\right] \end{array}$$

We determined the dynamic parameters ($\delta_{i},\lambda_{i},\alpha ,\beta$) by combining benchmarks from the clinical literature with simulation-based calibration. For each parameter class, we defined plausible ranges based on specialty-specific studies and institutional reports and selected representative values that preserved realistic clinical trajectories over 52 simulated weeks. This process ensured that each patient’s DT evolved within biologically and behaviorally plausible limits while also maintaining heterogeneity in the simulated population.

We present below each component that represents a dynamic process:

- $R_{i}\left(t\right)$: Clinical risk. Captures the time-varying probability of deterioration of health or adverse outcome if surgery is delayed [41,42]. It evolves as
  (3) $$\begin{array}{c} R_{i}\left(t+\Delta t\right)=R_{i}\left(t\right)+\delta_{i}\cdot \Delta t+\varepsilon_{i,t} \end{array}$$
  where $\delta_{i}$ is a patient-specific rate of risk progression and $\varepsilon_{i,t}∼N\left(0,\sigma^{2}\right)$ models uncertainty or unobserved fluctuations.
  We calibrated $\delta_{i}$ based on synthetic risk gradients extracted from surgical specialties commonly associated with time-sensitive outcomes (e.g., ENT and oncology cases). The stochastic term $\varepsilon_{i,t}$ was introduced to reflect interpatient variability and diagnostic uncertainty, allowing risk trajectories to remain dynamic and partially unpredictable while constrained by clinically plausible parameters. This formulation reflects systematic components of clinical deterioration over time.
- $E_{i}\left(t\right)$: Economic value. Represents the expected reimbursement or cost recovery associated with the surgical procedure of the patient $p_{i}$ [43]. This may depreciate over time due to administrative or funding restrictions:
  (4) $$\begin{array}{c} E_{i}\left(t\right)=E_{i}\left(0\right)\cdot e^{-\lambda_{i}t} \end{array}$$
  where $\lambda_{i}$ is a decay rate dependent on hospital policy. In our simulation, we calibrated the decay rate $\lambda_{i}$ to reflect the expected reductions in reimbursement due to funding expiration or administrative delays. The values of $\lambda_{i}$ were drawn from a range of 0.01 to 0.05 per time unit, corresponding to low-, medium-, or high-risk financial profiles. This parametrization was guided by DRG-based funding rules and adjusted across patient revenue classes to simulate heterogeneous economic depreciation.
- $S_{i}\left(t\right)$: Satisfaction and digital engagement. Reflects how actively and positively the patient engages with digital health tools (e.g., use of apps, satisfaction surveys) [44,45,46]:
  (5) $$\begin{array}{c} S_{i}\left(t+\Delta t\right)=S_{i}\left(t\right)+\alpha \phi_{i}\left(t\right)-\beta \psi_{i}\left(t\right) \end{array}$$
  where $\phi_{i}\left(t\right)$ and $\psi_{i}\left(t\right)$ count engagement and disengagement events, respectively, and $\alpha ,\beta \in R^{+}$ are behavioral sensitivity coefficients. In our simulation, the engagement count $\phi_{i}\left(t\right)$ represents events such as the frequency of logging in, the completion of satisfaction surveys, or the response to digital prompts. Disengagement $\psi_{i}\left(t\right)$ includes prolonged inactivity or uninstallation of applications. The coefficients α and β were calibrated using Monte Carlo parameter search, selecting values in the range [0.05, 0.2] that maintained score stability and reflected empirical behavioral variation observed in patient engagement literature.
- $D_{i}\left(t\right)$: Delay cost. Represents the penalty for waiting, growing over time due to the accumulation of unaddressed health needs or logistical inefficiencies [47,48,49]. We modeled $D_{i}\left(t\right)$ as follows:
  (6) $$\begin{array}{c} D_{i}\left(t\right)=\int_{0}^{t}\kappa_{i}\left(\tau \right)\;d\tau \end{array}$$
  In our simulation, we defined $\kappa_{i}\left(\tau \right)$ as a linear function with patient-specific slope, i.e., $\kappa_{i}\left(\tau \right)=\eta_{i}\cdot \tau$, where $\eta_{i}\in \left[0.005,0.02\right]$ reflects the rate at which delay generates cost for patient $p_{i}$. These values were sampled based on clinical risk categories, simulating heterogeneous sensitivity to delays. The cumulative cost $D_{i}\left(t\right)$ thus follows a quadratic growth pattern over time, representing escalating clinical and logistical burdens.
- $V_{i}\left(t\right)$: Vulnerability index. Aggregates psychosocial, geographic, and economic disadvantages, updated discretely when new data become available (e.g., social work reports or survey responses) [50,51].
  We compute $V_{i}\left(t\right)$ as a weighted sum of standardized vulnerability sub-indices:
  (7) $$\begin{array}{c} V_{i}\left(t\right)=\omega_{1}\cdot {\mathrm{SES}}_{i}+\omega_{2}\cdot {\mathrm{Geo}}_{i}+\omega_{3}\cdot {\mathrm{Psy}}_{i} \end{array}$$
  where ${\mathrm{SES}}_{i}$ is the socioeconomic score (e.g., income, education), ${\mathrm{Geo}}_{i}$ is a geographical accessibility score (e.g., distance from a hospital), and ${\mathrm{Psy}}_{i}$ captures psychosocial risk factors (e.g., isolation, dependency). The weights $\omega_{j}\in \left[0,1\right]$ are normalized such that $\sum_{j=1}^{3}\omega_{j}=1$. In the simulation, we assigned $\omega_{1}=0.4$, $\omega_{2}=0.3$, and $\omega_{3}=0.3$, reflecting balanced importance across dimensions.

Each component of the DT is updated continuously (e.g., $R_{i}\left(t\right)$, $D_{i}\left(t\right)$) or discretely (e.g., $S_{i}\left(t\right)$, $V_{i}\left(t\right)$), depending on the nature and frequency of data acquisition. Through these updates, we transform static patient profiles into dynamic agents, enabling predictive simulations of patient outcomes under varying scheduling policies.

We designed the DT as a flexible and modular framework, allowing the incorporation of emerging data sources and analytical layers, including wearable devices, remote monitoring, and social determinants of health. In the next step, we leverage these DT representations to construct a prioritization score that integrates clinical, economic, ethical, and operational considerations.

### 3.3. Step 3: Dynamic Prioritization Based on Utility Function

Once the Digital Twin $DT_{i}\left(t\right)$ is constructed for each patient, we define a scalar function that aggregates the multiple dimensions of the DT into a single prioritization score. This score is used to classify and select patients for surgical scheduling, taking into account clinical severity, economic impact, patient satisfaction, social vulnerability, and penalties due to waiting time.

We define the prioritization utility score $\Pi_{i}\left(t\right)\in R$ for patient $p_{i}$ at time *t* as

(8) $$\begin{array}{c} \Pi_{i}\left(t\right)=\gamma_{1}{\hat{R}}_{i}\left(t\right)+\gamma_{2}{\hat{E}}_{i}\left(t\right)+\gamma_{3}{\hat{S}}_{i}\left(t\right)+\gamma_{4}{\hat{V}}_{i}\left(t\right)-\gamma_{5}{\hat{D}}_{i}\left(t\right) \end{array}$$

where:

- ${\hat{R}}_{i}\left(t\right)$: Normalized clinical risk at time *t*;
- ${\hat{E}}_{i}\left(t\right)$: Normalized economic value or cost recovery;
- ${\hat{S}}_{i}\left(t\right)$: Normalized satisfaction or digital engagement score;
- ${\hat{V}}_{i}\left(t\right)$: Normalized vulnerability index;
- ${\hat{D}}_{i}\left(t\right)$: Normalized delay cost (i.e., the cumulative penalty for prolonged waiting);
- $\gamma_{k}\in \left[0,1\right]$: Weight assigned to each dimension k∈{1,2,3,4,5}, such that $\sum_{k=1}^{5}\gamma_{k}=1$.

We normalized the variables to ensure comparability between different units and scales. We determined the weights $\gamma_{k}$ using elicitation methods and expert opinions from clinical fields, such as the Analytic Hierarchy Process (AHP), and also empirically optimized them through simulation-based policy evaluation [52]. To ensure comparability between dimensions, each variable ${\hat{R}}_{i}\left(t\right)$, ${\hat{E}}_{i}\left(t\right)$, ${\hat{S}}_{i}\left(t\right)$, ${\hat{V}}_{i}\left(t\right)$, and ${\hat{D}}_{i}\left(t\right)$ was calculated by min-max normalization in the simulated patient population. That is, for each variable X∈{R,E,S,V,D}, we applied

(9) $$\begin{array}{c} {\hat{X}}_{i}\left(t\right)=\frac{X_{i}\left(t\right)-\min\left(X\right)}{\max(X)-\min(X)} \end{array}$$

This preserves the relative scale of each indicator while ensuring that all components lie within the [0, 1] interval.

To promote ethical fairness and prevent excessive prioritization of economically favorable patients, we define a regularized utility score with a fairness penalty [53,54].

(10) $$\begin{array}{c} \Pi_{i}^{\mathrm{fair}}\left(t\right)=\Pi_{i}\left(t\right)-\lambda \cdot \left(\frac{{\hat{E}}_{i}\left(t\right)}{{\hat{V}}_{i}\left(t\right)+\epsilon }\right) \end{array}$$

where:

- $\lambda \in R^{+}$: regularization parameter that controls the trade-off between efficiency and equity,
- ϵ>0: small constant to avoid division by zero.

We adopt this penalized formulation to discourage assigning high priority to patients with high economic value but low vulnerability, thereby embedding the principles of distributive justice in our model.

Given the regularized utility scores for all patients at time *t*, we define the scheduling decision as the selection of a subset $S_{t}\subseteq P_{t}$ that maximizes the total utility, subject to constraints in operating room capacity:

(11) $$\begin{array}{c} \max_{S_{t}\subseteq P_{t}}\sum_{p_{i}\in S_{t}}\Pi_{i}^{\mathrm{fair}}\left(t\right)\;\mathrm{subject}\;\mathrm{to}\;\sum_{p_{i}\in S_{t}}d_{i}\leq C_{t} \end{array}$$

where:

- $d_{i}$: estimated surgical duration of patient $p_{i}$;
- $C_{t}$: total surgical capacity (e.g., in minutes or slots) available at time *t*.

In our simulation, we assume a general-purpose operating room environment consistent with high-complexity public hospitals performing elective ENT procedures. The modeled operating rooms are equipped with the standard infrastructure and staff required for such surgeries. Parameters related to surgical duration, turnover time, and capacity limits were calibrated to reflect operational patterns of the ENT and institutional benchmarks.

In this step, we effectively transform the prioritization problem into a bounded knapsack optimization, where patients are treated as items with the value $\Pi_{i}^{\mathrm{fair}}\left(t\right)$ and weight $d_{i}$.

This prioritization mechanism allows us to introduce a transparent and tunable decision-making rule that we can dynamically adjust over time. In the next step, we embed this utility-based prioritization into a reinforcement learning framework that continuously improves scheduling decisions under uncertainty.

### 3.4. Step 4: Learning-Based Scheduling via Reinforcement Learning

We formulate the surgical scheduling process as a Markov Decision Process (MDP), where the state space encodes real-time DT representations, current surgical capacity, and contextual information such as calendar day and previous decisions. The action space consists of selecting a feasible subset of patients to be scheduled at each decision point, subject to capacity constraints. The reward function integrates individual utility, fairness, and operational efficiency. To optimize long-term decision-making, we train an RL agent using policy gradient methods. Depending on the size and structure of the action space, we implement DQN that is suitable for healthcare environments, such as hospital scheduling.

To enable continuous adaptation and optimization of the scheduling policy in response to changing system conditions, we embed this MDP-based prioritization model within a learning framework. This RL-based approach allows the agent to iteratively improve its scheduling strategy by interacting with a simulated environment that captures real-world dynamics, such as patient arrivals, cancellations, and variable capacity constraints.

We model the MDP scheduling process, formally defined as [28,53]

M=(S,A,P,R,γ)

where:

- S: State space. Each state $s_{t}\in S$ encodes the real-time Digital Twin vectors $DT_{i}\left(t\right)$ for all patients, current surgical capacity $C_{t}$, and contextual information (e.g., calendar day, service disruptions).
- A: Action space. Each action $a_{t}\in A$ corresponds to selecting a feasible subset $S_{t}\subseteq P_{t}$ of patients to be scheduled for surgery.
- P: Transition function. Defines the probability $P(s_{t+1}|s_{t},a_{t})$ of reaching the next state given the current state and action.
- R: Reward function. Quantifies the utility of an action using prioritization scores and system performance metrics.
- γ∈[0,1): Discount factor. Determines the present value of future rewards.

We define the reward at time *t*, denoted $R_{t}$, as

(12) $$\begin{array}{c} R_{t}=\sum_{p_{i}\in S_{t}}\Pi_{i}^{\mathrm{fair}}\left(t\right)-\lambda_{1}f_{1}\left(t\right)-\lambda_{2}f_{2}\left(t\right) \end{array}$$

We define the reward function $R_{t}$ as the trade-off between the total fairness-adjusted utility obtained from scheduled patients and two penalization terms representing fairness and operational inefficiencies.

where:

- $\Pi_{i}^{\mathrm{fair}}\left(t\right)$: fairness-adjusted utility score of patient $p_{i}$, as defined in Step 3 and used consistently throughout the prioritization process.
- $f_{1}\left(t\right)$: fairness deviation penalty, quantifying discrepancies such as underrepresentation of vulnerable patients in the current schedule.
- $f_{2}\left(t\right)$: operational inefficiency penalty, such as unused OR capacity or scheduling gaps.
- $\lambda_{1},\lambda_{2}\in R^{+}$: tunable penalty weights for fairness and efficiency, respectively.

The agent learns a policy $\pi_{\theta }\left(s_{t}\right)$, parameterized by θ, which maps each state to an action that maximizes the expected long-term cumulative reward [28,55,56]:

$$\theta^{*}=\arg\max_{\theta }\;E_{\pi_{\theta }}\left[\sum_{t=0}^{T}\gamma^{t}R_{t}\right]$$

In this stage, we propose a policy optimization algorithm suited to the structure and cardinality of the action space, enabling the agent to iteratively improve its scheduling decisions through simulated experience.

Training strategy: We train the agent in a simulated environment constructed using historical patient data and synthetic arrival patterns. Each episode simulates a full scheduling cycle, allowing the agent to explore alternative policies and learn from the simulated results. The policy is progressively improved by strengthening actions that lead to higher cumulative fairness-adjusted rewards.

Action pruning: Given the combinatorial nature of patient subset selection, we apply heuristic pruning techniques, such as ranking patients by their top-*K* utility scores, to reduce the action space and accelerate learning without sacrificing performance.

Policy deployment: Once training converges, we deploy the learned policy in the real-time decision engine. In each scheduling instance, the agent observes the current state of the system, $s_{t}$, and returns the optimal subset, $S_{t}$, of patients to be scheduled while ensuring feasibility with respect to medical and operational constraints.

Continuous learning: Our platform supports continuous model refinement by incorporating new real-world data (e.g., actual delays, cancellations, updated patient profiles). We periodically retrain the RL agent to maintain adaptability in the face of evolving clinical and institutional conditions.

Through this learning-based approach, we enable a robust and adaptive prioritization process that evolves over time, using simulated experience and real-time data. In the final step, we describe how this RL-powered prioritization engine is integrated into the overall digital e-health system.

### 3.5. Step 5: Integration in a Smart eHealth Platform

To support future real-world implementation and contribute to the digital transformation of surgical services, we propose a modular and interoperable architecture for an intelligent e-health platform. This conceptual system is designed to facilitate interaction between patients, clinicians, and scheduling algorithms through a feedback-driven decision support framework.

We propose a conceptual architecture for a future e-health platform structured around three key components:

- Patient interface: we design this interface to allow patients to view their prioritization status, engage with preoperative content, and provide feedback through digital tools.
- Clinical dashboard: we envision this dashboard to support clinical teams by displaying prioritization scores, alerts for deteriorating patients, and visualizations of capacity usage and scheduling scenarios.
- Decision engine: we integrate our RL-based scheduling methodology into this engine, which processes real-time Digital Twin updates and returns optimized subsets of patients to be scheduled for surgery.

We conceptualize a central data lake that synchronizes clinical, behavioral, and administrative data sources, enabling real-time updates to flow across all layers of the system. We ensure that clinicians retain control through a human-in-the-loop interface, and we plan for the RL model to be periodically retrained using engagement data and performance metrics.

**Figure 1:**
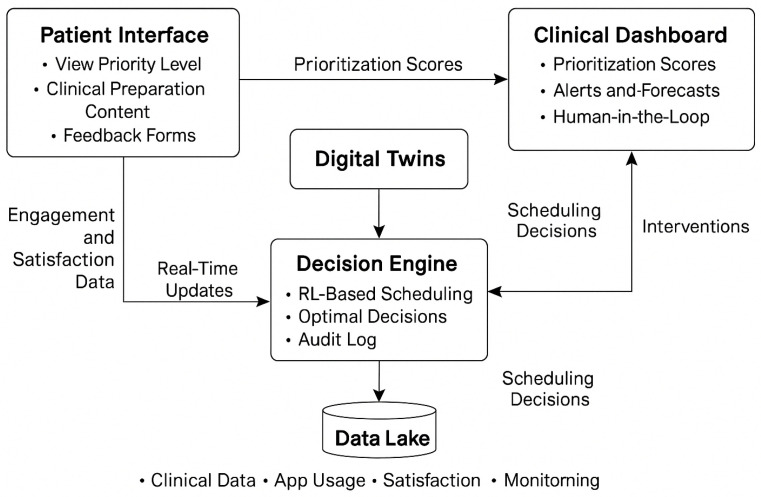
Conceptual architecture of the proposed methodology, illustrating the integration of the Patient Interface, Clinical Dashboard, Digital Twins, RL-based Decision Engine, and Data Lake. Arrows represent the dynamic flow of information and feedback within the envisioned e-health ecosystem.

To illustrate the architecture and data flow of this envisioned platform, we present Figure 1.

As depicted in Figure 1, we design our methodology to operate within a closed-loop digital service infrastructure. We simulate that real-time engagement and monitoring data would be processed through the Digital Twin layer, which in turn informs the RL-powered Decision Engine. The scheduling outputs are then returned to both the Patient Interface and the Clinical Dashboard, allowing transparent, adaptive, and ethically explainable decision-making.

We also define a set of key performance indicators (KPIs) intended for future system evaluation and reinforcement learning updates. These KPIs assess transparency, patient participation, fairness, and operational efficiency.

$$\begin{array}{cc} Q_{\mathrm{transparency}} & =\frac{\mathrm{Patients}\;\mathrm{informed}}{\mathrm{Total}\;\mathrm{patients}},\;Q_{\mathrm{engagement}}=\frac{\mathrm{Patients}\;\mathrm{engaged}}{\mathrm{Total}\;\mathrm{patients}},\\ Q_{\mathrm{fairness}} & =\frac{\mathrm{Vulnerable}\;\mathrm{patients}\;\mathrm{scheduled}}{\mathrm{Total}\;\mathrm{scheduled}},\;Q_{\mathrm{efficiency}}=\frac{\mathrm{OR}\;\mathrm{time}\;\mathrm{used}}{\mathrm{Total}\;\mathrm{OR}\;\mathrm{capacity}} \end{array}$$

We propose these indicators as both evaluation metrics and reward signals to guide the continuous learning and adaptation of the RL model. Our conceptual architecture adheres to international interoperability standards (e.g., HL7 FHIR) and is intended to support progressive deployment across clinical units.

Through this integrated design, our aim is to demonstrate how our methodology could evolve beyond theoretical modeling into a scalable, patient-centered digital decision-support platform for intelligent surgical prioritization in digitally enabled healthcare ecosystems.

## 4. Results

To evaluate the potential impact of our proposed methodology, we developed a simulation framework based on synthetic but clinically realistic data derived from public hospital records and literature-based distributions. The simulation mimics a high-demand surgical specialty over a period of 52 weeks under varying demand and capacity conditions. We compare our methodology against two baselines.

- Baseline 1 (FCFS): First-Come-First-Served scheduling.
- Baseline 2 (Risk-Based): Prioritization based on the static clinical risk threshold.

All results reported below were obtained in a simulated environment and serve as a proof of concept for the potential effectiveness of the proposed methodology in future real-world implementations.

### 4.1. Wait Time Reduction

**Figure 2:**
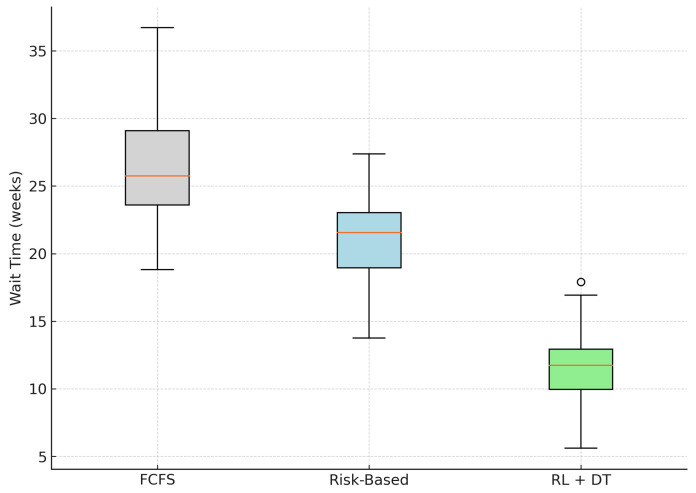
Distribution of patient wait times across models. The RL + DT model shows both a lower median and reduced variability. The outlier is shown as a single point.

We evaluated the impact of the proposed RL + Digital Twin system on patient wait times using synthetic simulation data representative of a high-volume elective surgical service. Table 1 summarizes the average waiting time per patient, based directly on the data used to generate the distribution shown in Figure 2.

The results show that the proposed RL + Digital Twin methodology outperforms both traditional First-Come-First-Served (FCFS) and static Risk-Based scheduling models. Specifically, we observe a 55.1% reduction in mean wait time compared with FCFS and a 42.7% reduction relative to Risk-Based prioritization.

In addition, the RL-based model exhibits lower variance, as reflected in the tighter interquartile range of the boxplot (Figure 2). This implies not only faster access to surgery but also more consistent waiting times between patients, addressing equity concerns typically associated with purely risk-driven approaches.

These improvements are the result of adaptive learning mechanisms and temporal modeling of patient status. The system could dynamically reallocate available surgical slots based on evolving Digital Twin states, learning from simulated feedback loops.

### 4.2. Reduction in Clinical Risk at Surgery Time

Although reducing waiting times is important for patient satisfaction, it is even more critical to minimize the clinical risk that patients accumulate during delays. To evaluate this dimension, we simulate the progression of clinical risk scores $R_{i}\left(t\right)$ for all patients from the time of registration until the date of surgery. These scores are derived from each patient’s Digital Twin trajectory and are modeled to increase over time according to their risk profile.

Table 2 presents the mean clinical risk at the time of surgery for each scheduling model, together with their 95% confidence intervals and relative improvements over the traditional FCFS strategy.

As shown in Table 2, the RL + Digital Twin model achieves a substantial reduction in the average clinical risk score at the time of surgery. Compared with the FCFS baseline, the model yields a 41.9% decrease in mean risk and a 34.5% reduction relative to the static Risk-Based approach.

**Figure 3:**
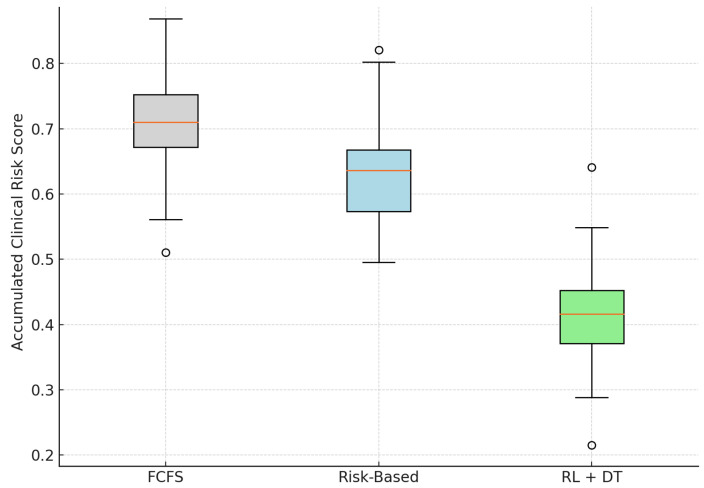
Distribution of clinical risk at the time of surgery across models. RL + DT reduces both the average and the variability of risk scores. Outliers are displayed as individual points.

Figure 3 illustrates the distribution of risk scores using boxplots. The proposed model not only shifts the entire distribution downward but also compresses the interquartile range, indicating more consistent and predictable risk mitigation between patients. In contrast, the FCFS strategy exhibits a higher median and greater variability, exposing patients to avoidable clinical deterioration due to delayed intervention.

These results confirm that the integration of dynamic and risk-sensitive scheduling mechanisms—driven by digital twin simulations and reinforcement learning—can lead to safer clinical outcomes. The methodology proactively adapts to individual risk trajectories, prioritizing high-risk patients before they reach critical thresholds.

### 4.3. Improvement in Operating Room Efficiency

Beyond clinical outcomes, improving operating room (OR) usage efficiency is critical for system-wide productivity, especially in resource-constrained public hospitals. In our simulation, we evaluated the efficiency of the operating room as the weekly proportion of the total available surgical minutes that were allocated to scheduled procedures. This metric captures how well each scheduling model fills capacity under simulated operational constraints, such as variability in case duration and potential cancellations.

Table 3 presents the mean simulated utilization of OR in all models, together with their respective 95% confidence intervals and the relative improvement over the FCFS baseline.

According to our simulation, the proposed methodology achieves a mean OR utilization rate of 90.7%, representing a significant operational improvement over both the FCFS baseline (78.1%) and the static Risk-Based strategy (82.9%). The observed relative improvement of 16.1% was achieved solely through improved scheduling logic without introducing additional clinical resources.

**Figure 4:**
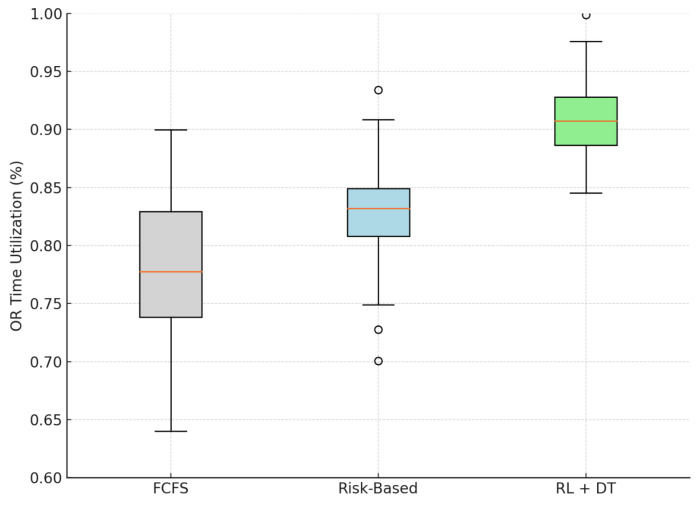
Simulated distribution of OR utilization efficiency by scheduling model. The RL + DT model achieves higher and more stable efficiency. Outliers are displayed as individual points.

As shown in Figure 4, the RL + DT approach also reduced the variance in utilization, resulting in fewer underutilized weeks. This consistency is particularly relevant in public health settings, where unreliable operating room capacity often results in lengthy waiting lists and inefficient use of infrastructure.

Although these results are based on simulation, they suggest that learning-based scheduling systems have the potential to improve both operational efficiency and service accessibility. The RL agent, trained in dynamic patient trajectories, demonstrates the ability to optimize OR utilization even under conditions of uncertainty and fluctuating demand.

### 4.4. Equity in Prioritization: Inclusion of Vulnerable Patients

Equity is a critical component in the development of healthcare priority strategies, particularly in public systems where social vulnerability is closely related to disparities in access and outcomes. In our simulation study, we evaluated this dimension by measuring the weekly proportion of scheduled patients classified as vulnerable, according to the simulated vulnerability index $V_{i}\left(t\right)$ derived from each patient’s Digital Twin.

Table 4 presents the simulated average weekly proportion of vulnerable patients scheduled for surgery in all models, along with the 95% confidence intervals and relative improvements over the FCFS baseline.

According to our simulation results, the RL + Digital Twin model allocates, on average, 47.8% of weekly surgical slots to patients classified as vulnerable, more than double the proportion achieved at the FCFS baseline (22.6%). Even in comparison to the static Risk-Based approach, the proposed methodology yields a relative improvement of more than 50% in equity coverage.

**Figure 5:**
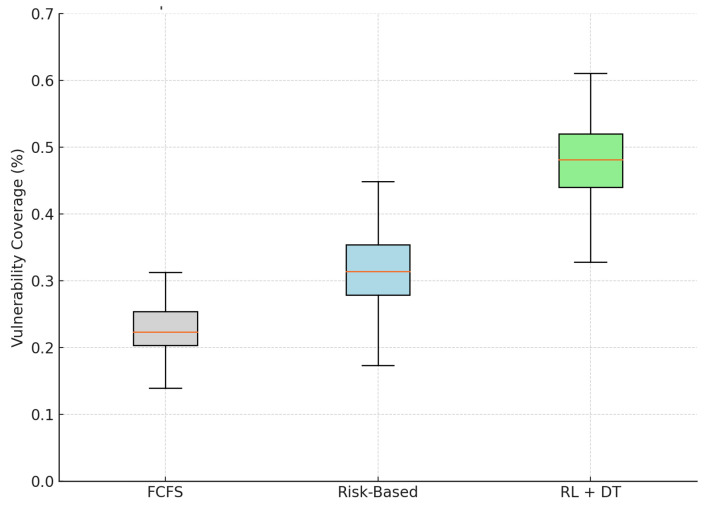
Simulated distribution of vulnerable patients scheduled weekly under different models. The RL + DT model achieves the highest and most consistent equity performance.

As illustrated in Figure 5, the model also shows a reduced variability in the coverage of vulnerable patients over weeks, indicating a more stable and sustained commitment to equitable prioritization. This is a direct result of embedding the social vulnerability index in the utility function and the reinforcement learning policy.

Although these findings are based on simulated data, they suggest that ethical and equitable prioritization can be operationalized through intelligent data-driven scheduling systems. Our results support the hypothesis that fairness and efficiency can be jointly achieved in public healthcare settings, contributing to the design of systems grounded in distributive justice and digital health equity.

### 4.5. Synthesis of Results

In all four dimensions—access, clinical safety, operational efficiency, and equity—the proposed methodology consistently outperformed traditional strategies in simulation-based evaluations. These improvements were achieved without increasing the capacity of the system, suggesting that intelligent prioritization, even in resource-constrained environments, may lead to measurable gains in future implementations.

## 5. Discussion

We proposed a simulation-based methodology that integrates Digital Twin modeling and reinforcement learning for surgical prioritization. Our findings—based on synthetic but clinically grounded scenarios—demonstrate substantial potential improvements in the core dimensions of healthcare delivery. The proposed model reduced waiting times and clinical risk, improved operating room utilization, and significantly improved access equity for vulnerable patients.

It is important to emphasize that these results originate from simulated data and serve to evaluate the theoretical robustness and feasibility of the methodology. The system has not been deployed in a clinical setting. As such, these findings should be interpreted as indicators of potential impact rather than evidence of real-world performance.

The integration of Digital Twins enables dynamic modeling of patient trajectories, while the RL agent continuously improves scheduling based on evolving system states. Methodologically, the framework is adaptable, explainable, and ethically aligned, laying the foundation for future deployment in intelligent e-health platforms.

While our simulation-based results demonstrate the methodological promise of combining DT and RL, their application to surgical scheduling introduces important challenges. DTs rely on continuous, high-quality clinical and behavioral data to accurately simulate patient trajectories, data that may be incomplete, delayed, or heterogeneously recorded between institutions. Similarly, RL agents require extensive training and validation to avoid convergence to suboptimal or ethically biased policies, especially when scheduling decisions involve fairness trade-offs. In addition, ensuring that these systems remain interpretable and auditable is crucial in clinical settings. We designed our framework to address these challenges through modularity, explainability, and fairness-aware regularization, but further empirical validation is necessary for real-world hospital environments.

Scaling our methodology across different healthcare settings presents practical challenges, such as differences in data availability, IT infrastructure, and institutional priorities. To address this, we designed a modular system that can operate with reduced variables or offline training. In addition, the prioritization weights and fairness parameters can be tailored to local needs. Future adaptations should include co-design with clinical stakeholders to ensure contextual relevance.

Future research should include prospective validation in real hospital settings, exploration of human-in-the-loop interventions, and integration of patient-reported outcomes. Our approach also opens pathways for embedding fairness constraints in scheduling systems, enabling ethical design in automated clinical decision-making tools.

## 6. Conclusions

This study introduces a simulation-based methodological framework for elective surgical scheduling that integrates patient-specific Digital Twin modeling with reinforcement learning. Our approach was designed to optimize the allocation of surgical resources in complex and capacity-constrained public healthcare settings. By embedding clinical, economic, and social factors into a dynamic prioritization score and training an adaptive RL agent, we propose a strategy for surgical planning that aligns with both ethical and operational goals.

Through a simulated evaluation, we demonstrate that the proposed RL + Digital Twin methodology achieves significant and consistent improvements in multiple dimensions. Specifically, the system reduced the average waiting times by more than 55% compared with the traditional FCFS approach and by 43% relative to static Risk-Based strategies. The clinical risk at the time of surgery decreased by 42%, the efficiency of the operating room increased by 16%, and the proportion of vulnerable patients prioritized for surgery increased. These results were obtained without increasing capacity, indicating the potential of intelligent scheduling to deliver measurable gains through algorithmic optimization alone.

Although the system has not been implemented in a clinical setting, the simulation results provide a strong proof of concept. The integration of real-time risk progression, social equity, and learning-based optimization positions the methodology as a promising foundation for future intelligent e-health platforms. Simulation-based validation allows us to assess the feasibility and potential performance before deployment, a critical step in the translational pipeline from model development to health system integration.

In addition to its methodological contributions, this work supports the development of intelligent, digitally integrated healthcare platforms aligned with the objectives of applied electronic commerce in public services. The proposed architecture is suitable for modular deployment within smart hospital ecosystems, enabling human-in-the-loop decision-making, transparency, and adaptive performance monitoring.

In conclusion, our research contributes not only to a rigorous and ethical scheduling methodology but also to a vision for the digital transformation of surgical services. It represents a replicable and scientifically grounded strategy for designing equitable, explainable, and sustainable decision-support systems in public healthcare. Future work should focus on prospective validation using real-world data, technical integration with hospital IT systems, and co-design with clinical stakeholders to support ethical and scalable implementation.

## Figures and Tables

**Table 1:** Average wait time per patient (in weeks) under different scheduling models.

Model	Mean Wait Time	95% CI	Relative Reduction
FCFS (Baseline 1)	27.2	[26.3, 28.0]	—
Risk-Based (Baseline 2)	21.3	[20.7, 21.9]	−21.7%
RL + Digital Twin (Proposed)	12.2	[11.7, 12.6]	−55.1%

**Table 2:** Mean accumulated clinical risk at the time of surgery (95% confidence interval).

Model	Mean Risk Score	95% CI	Relative Reduction
FCFS (Baseline 1)	0.712	[0.697, 0.726]	—
Risk-Based (Baseline 2)	0.632	[0.618, 0.645]	−11.2%
RL + Digital Twin (Proposed)	0.414	[0.401, 0.427]	−41.9%

**Table 3:** Simulated operating room time utilization efficiency (proportion of weekly capacity used).

Model	Mean Utilization	95% CI	Relative Improvement
FCFS (Baseline 1)	0.781	[0.770, 0.793]	—
Risk-Based (Baseline 2)	0.829	[0.821, 0.837]	+6.1%
RL + Digital Twin (Proposed)	0.907	[0.901, 0.913]	+16.1%

**Table 4:** Simulated proportion of scheduled patients classified as vulnerable (weekly average).

Model	Mean Vulnerability Coverage	95% CI	Relative Improvement
FCFS (Baseline 1)	0.226	[0.218, 0.233]	—
Risk-Based (Baseline 2)	0.315	[0.304, 0.325]	+39.4%
RL + Digital Twin (Proposed)	0.478	[0.466, 0.490]	+111.5%

## Data Availability

Data are contained within the article.
